# Rapid, robust, and sustainable antibody responses to mRNA COVID-19 vaccine in convalescent COVID-19 individuals

**DOI:** 10.1172/jci.insight.151477

**Published:** 2021-10-22

**Authors:** Sabrina E. Racine-Brzostek, Jim K. Yee, Ashley Sukhu, Yuqing Qiu, Sophie Rand, Paul D. Barone, Ying Hao, He S. Yang, Qing H. Meng, Fred S. Apple, Yuanyuan Shi, Amy Chadburn, Encouse Golden, Silvia C. Formenti, Melissa M. Cushing, Zhen Zhao

**Affiliations:** 1 Department of Pathology and Laboratory Medicine, Weill Cornell Medicine, New York, New York, USA.; 2 NewYork-Presbyterian Hospital, Weill Cornell Medical Campus, New York, New York, USA.; 3 Department of Population Health Sciences, Weill Cornell Medicine, New York, New York, USA.; 4 School of Life Sciences, Beijing University of Chinese Medicine, Beijing, China.; 5 Department of Laboratory Medicine, The University of Texas MD Anderson Cancer Center, Houston, Texas, USA.; 6 Departments of Laboratory Medicine and Pathology at Hennepin Healthcare/Hennepin County Medical Center and University of Minnesota, Minneapolis, Minnesota, USA.; 7 Department of Biomedical Engineering, Shenzhen Research Institute, Beijing University of Chinese Medicine, Shenzhen, China.; 8 Department of Radiation Oncology and; 9 Department of Medicine, Weill Cornell Medicine, New York, New York, USA.

**Keywords:** COVID-19, Immunology, Adaptive immunity

## Abstract

Longitudinal studies are needed to evaluate the SARS-CoV-2 mRNA vaccine antibody response under real-world conditions. This longitudinal study investigated the quantity and quality of SARS-CoV-2 antibody response in 846 specimens from 350 patients, comparing BNT162b2-vaccinated individuals (19 previously diagnosed with COVID-19, termed RecoVax; and 49 never diagnosed, termed NaiveVax) with 122 hospitalized unvaccinated (HospNoVax) and 160 outpatient unvaccinated (OutPtNoVax) COVID-19 patients. NaiveVax experienced delay in generating SARS-CoV-2 total antibodies (TAb) and surrogate neutralizing antibodies (SNAb) after the first vaccine dose (D1) but rapid increase in antibody levels after the second dose (D2). However, these never reached RecoVax’s robust levels. In fact, NaiveVax TAb and SNAb levels decreased 4 weeks after D2. For the most part, RecoVax TAb persisted, after reaching maximal levels 2 weeks after D2, but SNAb decreased significantly about 6 months after D1. Although NaiveVax avidity lagged behind that of RecoVax for most of the follow-up periods, NaiveVax did reach similar avidity by about 6 months after D1. These data suggest that 1 vaccine dose elicits maximal antibody response in RecoVax and may be sufficient. Also, despite decreasing levels in TAb and SNAb over time, long-term avidity may be a measure worth evaluating and possibly correlating to vaccine efficacy.

## Introduction

As the COVID-19 pandemic enters its second year with more than 193 million confirmed cases worldwide (1), many countries look to effective prophylactic SARS-CoV-2 vaccines to help curb its spread and prevent the thousands of COVID-19 deaths reported daily (2). In December 2020, the US Food and Drug Administration (FDA) issued an emergency use authorization (EUA) for 2 SARS-CoV-2 mRNA vaccines. Shortly thereafter, New York state commenced its vaccination program by vaccinating health care workers.

In addition to clinical trials conducted to determine the safety and efficacy of the mRNA vaccines (3–5), studies began describing the serological response to the vaccines under “real-world” conditions, coinciding with the onset of the SARS-CoV-2 variants and case reports of vaccine escape (6–13). Although the initial focus may be on overall antibody levels and differences in the antibody response in previously seropositive versus seronegative vaccine recipients (6, 8, 11), other humoral antibody response factors need to be considered. Due to their role in inactivating viruses and limiting the number of infected host cells, neutralizing antibodies are often considered a gold standard in evaluating protective immune responses (14). Early studies describe differences in the neutralizing response postvaccination in those previously exposed versus naive to SARS-CoV-2 (12, 15, 16). Binding avidity, the intrinsic affinity of the antibody-antigen interaction, is another potential factor in evaluating the quality of the antibody response. Studies show that over time, the low-avidity antibodies produced early in the humoral immune response to SARS-CoV-2 mature and strengthen, displaying higher intrinsic affinity (17–19). However, this has not been studied in SARS-CoV-2–vaccinated individuals to our knowledge.

This study evaluated the dynamics of the antibody response to the BNT162b2 mRNA SARS-CoV-2 vaccine, including total antibody (TAb) levels, neutralizing antibody levels, and antibody avidity, in 49 noninfected vaccinated (NaiveVax) and 19 previously infected vaccinated (RecoVax) health care workers. The vaccine-induced response was then compared with the natural postinfection antibody response in 160 nonvaccinated outpatients with mild COVID-19 symptoms (OutPtNoVax) and 122 nonvaccinated, hospitalized, acutely infected patients with COVID-19 (HospNoVax) (20) during the early period of the pandemic.

## Results

### Participant demographics

#### Vaccine study cohorts (prospective).

Participant demographics of the 68 health care worker volunteers who had been vaccinated December 18, 2020, to February 11, 2021, with the BNT162b2 vaccine are summarized in Table 1.

RecoVax cohort consisted of 19 participants (27.9%) previously diagnosed with symptomatic COVID-19 either by real-time reverse transcription PCR (RT-PCR) (7/19; 36.8%) or by prior serology (9/19; 47.4%) or clinically diagnosed with COVID-19 during the early periods of the pandemic when testing was unavailable (3/19; 15.8%). The median time from COVID-19 symptom onset to first dose of vaccine (D1) in this cohort was 262 days (IQR 102–275). All 19 participants tested positive for SARS-CoV-2 serology when participant sera were evaluated with the Roche Elecsys Anti-SARS-CoV-2 assay, which identifies nucleocapsid protein (N protein) antibodies produced by infection rather than vaccination because the BNT162b2 vaccine does not include the N protein.

**Figure 1:**
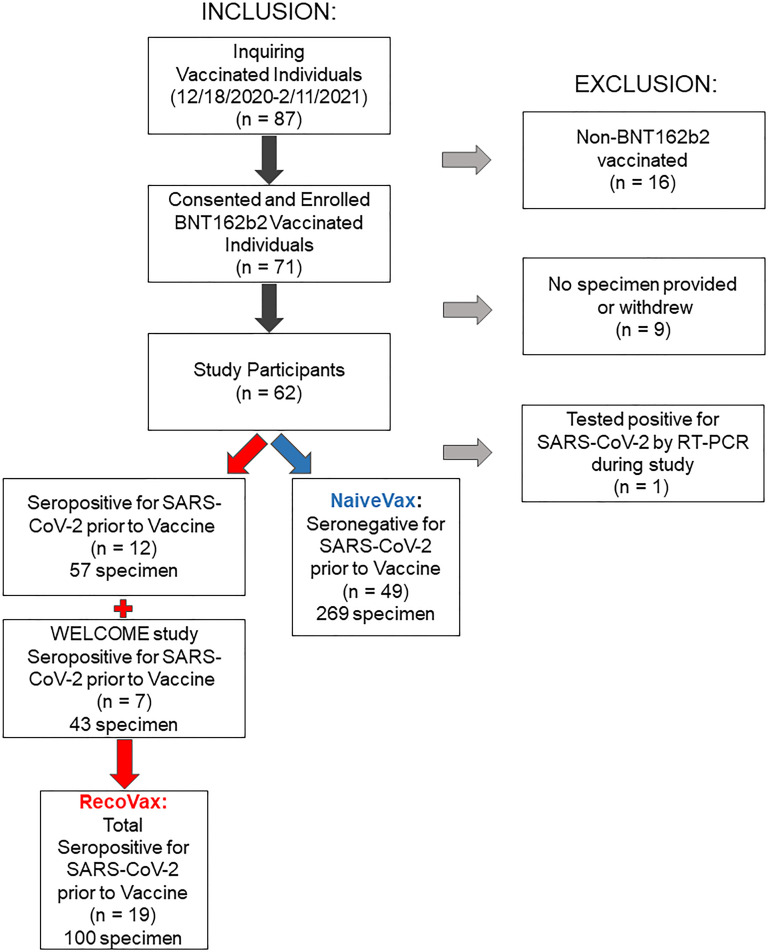
Inclusion and exclusion criteria of the prospective vaccination arm of the study.

NaiveVax cohort consisted of 50 participants, not diagnosed with COVID-19 and without antibodies against the N protein at the onset of the study. However, 1 participant was excluded due to testing positive for COVID-19 by RT-PCR 6 days after the first vaccine dose (Figure 1).

#### Nonvaccinated cohorts (retrospective).

HospNoVax cohort consisted of 122 adult patients who had presented to the emergency department (ED) and were subsequently hospitalized at NewYork-Presbyterian Hospital, Weill Cornell Medical Campus (NYP/WCMC), during the first month of the pandemic in New York City (March 8, 2020, to April 7, 2020). The antibodies generated by these patients would be most consistent with the initially described SARS-CoV-2. This comparison was prudent as multiple variants have since been described (21), and the vaccine’s design was based on the nucleoside-modified mRNA that encodes the trimerized receptor-binding domain (RBD) of the early SARS-CoV-2 spike glycoprotein (22). HospNoVax demographics are summarized in Table 1 and further described in a previous validation study (23). The median time from COVID-19 symptom onset to the ED visit was 7 days (IQR 4–10). In an attempt to estimate time of infection for comparison studies with the vaccinated cohorts, it was estimated that the time of infection was 5 days prior to date of symptom onset (24, 25). Therefore, the median estimated time of infection to the ED visit was 12 days (IQR 7–15). Also of note, 39/122 (32%) were intubated, and 32/122 (26.2%) died during their hospitalization (23).

OutPtNoVax cohort consisted of 160 adult patients who had presented to an outpatient clinic in person or via video visit and had been tested by serology on the basis of suspicion of prior COVID-19 infection. Specimen collection for SARS-CoV-2 serology testing occurred from April 30, 2020, to May 20, 2020. As with the hospitalized patients, antibodies generated by these patients would be most consistent with the initially described SARS-CoV-2. This outpatient cohort’s demographics are summarized in Table 1. The median time from COVID-19 symptom onset to the outpatient visit was 47 days (IQR 43–48). As with the hospitalized cohort, it was estimated that the time of infection was 5 days prior to date of symptom onset. Therefore, the median estimated time of infection to the outpatient visit was 52 days (IQR 48–53).

### Quantitative antibody response during the first 2 months postvaccination compared with postinfection

**Figure 2:**
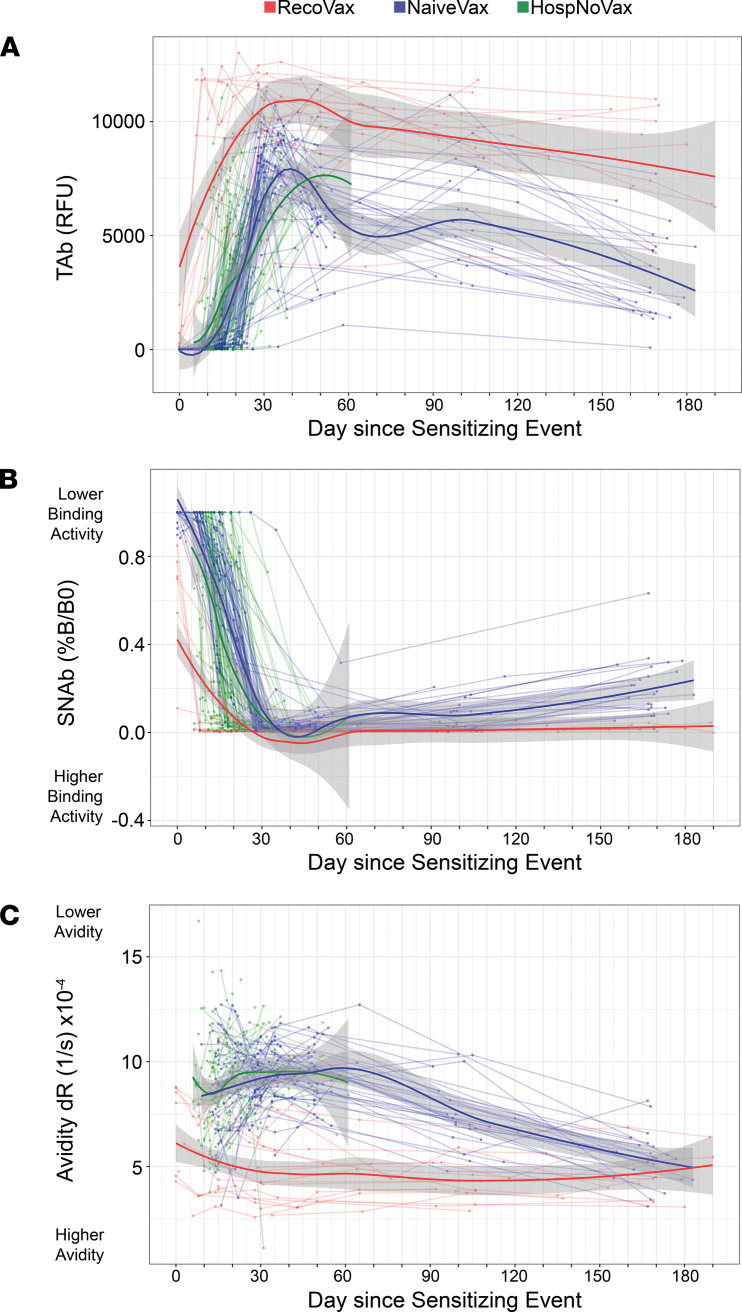
Dynamics of the anti–SARS-CoV-2 antibody response after vaccination or infection utilizing regression models. TAb (**A**) and SNAb (**B**) levels and avidity (**C**) are displayed over time. A total of 686 data points were plotted from 19 RecoVax individuals (shown in red), 49 NaiveVax individuals (blue), and 122 HospNoVax patients (green). All participants received the second dose 21 days after the first dose. The trend of antibody level over time was described by applying Muggeo’s method of estimating regression models with unknown breakpoints to estimate the changing time points of the trends. RFU, relative fluorescence units; SNAb, surrogate neutralizing antibodies; dR, relative dissociation rate.

The quantitative antibody responses between vaccinated and unvaccinated cohorts were examined using regression modeling (Figure 2). It was found that HospNoVax had a gradual rise in anti–SARS-CoV-2 TAb levels up to 33 days postinfection with the TAb RFU increasing 277 RFU/d (*P* < 0.001), at which point the levels began to plateau, and no significant change was observed to the last follow-up period of 61 days (mean 7003 RFU, coefficient 23, *P* = 0.735) (Figure 2A).

NaiveVax displayed a slight lag in responding after the first dose of the vaccine in comparison with RecoVax, but TAb levels increased at a similar rate to that of HospNoVax postinfection — at a rate of 280 RFU/d (*P* < 0.001). At approximately day 34 after D1, TAb began its slow overall decline at 29.4 RFU/d until the end of the follow-up period of up to 183 days after D1 (*P* < 0.001) (Figure 2A).

This is in stark contrast to the robust TAb response within the first week of vaccination in RecoVax. The TAb increased 1553 RFU/d for 7 days (*P* < 0.001), at which point the RFU levels began to plateau and then began to wane at a slower rate than NaiveVax at 14.72 RFU/d (*P* < 0.001) (Figure 2A).

**Figure 3:**
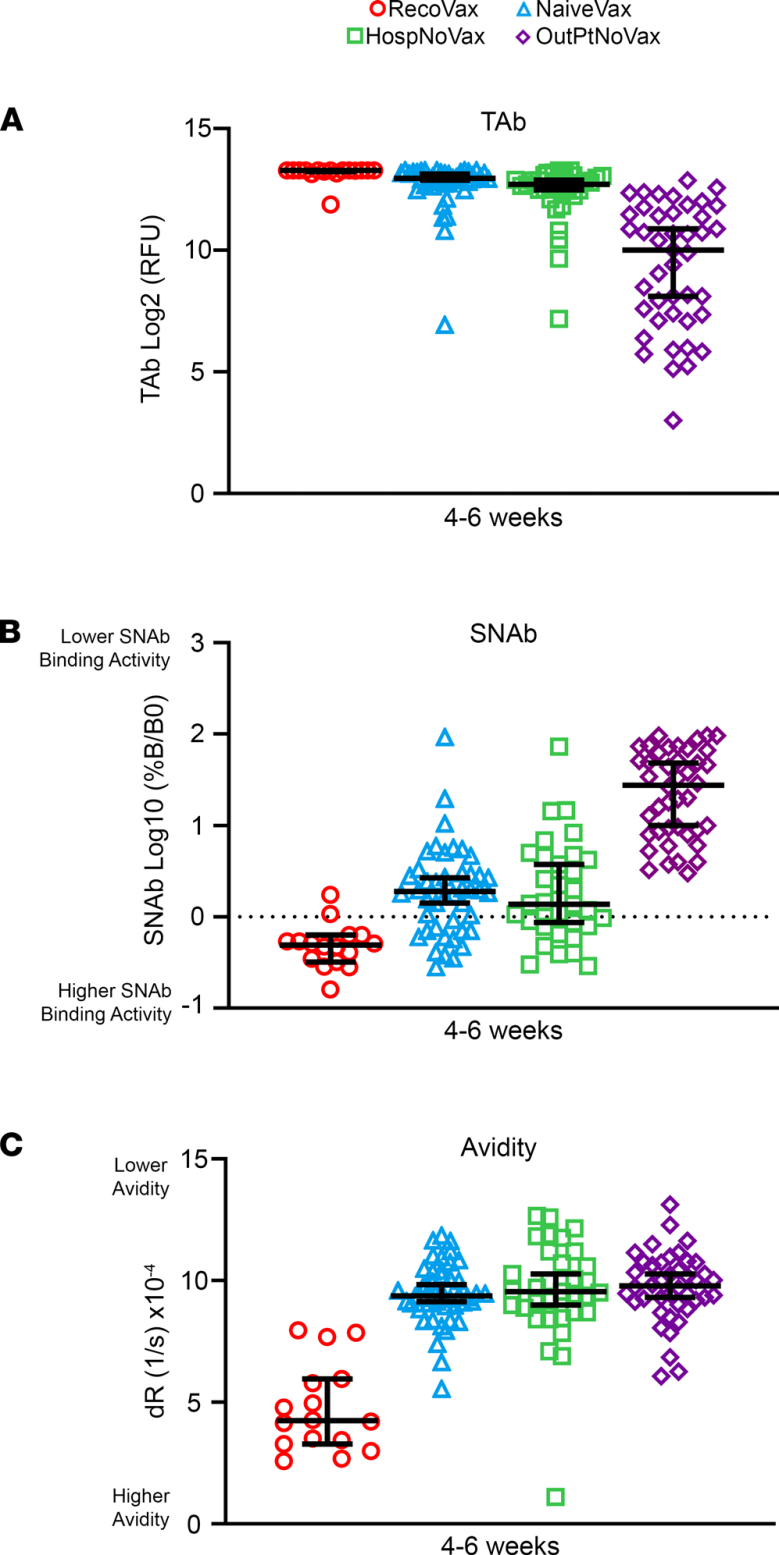
Comparison of SARS-CoV-2 antibody profiles of RecoVax, NaiveVax, HospNoVax, and OutPtNoVax cohorts 4–6 weeks after vaccination or infection. TAb antibody response (**A**), SNAb levels (**B**), and avidity (**C**) 4–6 weeks after vaccination or infection in RecoVax (red circle; *n* = 16), NaiveVax (blue triangle; *n* = 44), HospNoVax (green square; *n* = 43), and OutPtNoVax (violet diamond; *n* = 35) individuals. Horizontal black lines represent median values and whiskers represent 95% CI. Wilcoxon rank-sum was used for paired comparison while Kruskal-Wallis test was used for the comparison of 3 or more groups.

To complement the regression model, the TAb, neutralizing activity, and avidity levels in all 3 cohorts were analyzed by stratifying into three 2-week time periods: 0–13 days (0–2 weeks), 14–27 days (2–4 weeks), and 28–42 days (4–6 weeks) after infection or D1. Data are provided in Supplemental Figure 1; supplemental material available online with this article; https://doi.org/10.1172/jci.insight.151477DS1 Additional comparisons were made between the vaccinated cohorts and OutPtNoVax at 4–6 weeks and 6–8 weeks postinfection or D1. Data are provided in Supplemental Figure 2. Figure 3 displays the antibody response at 4–6 weeks postinfection or D1, a time period that was available for comparison in all 4 cohorts in the retrospective and prospective arms of the study.

Of note, RecoVax consistently had higher TAb levels than NaiveVax, HospNoVax, and OutPtNoVax during the first 1.5–2 months after vaccination or infection (Supplemental Figure 1A, Supplemental Figure 2A, and Figure 3A). The RecoVax TAb was substantially increased in the first 2 weeks after D1, with a median TAb of 882 RFU (IQR 93–9916), and continued to rise to a median TAb of 9916 (IQR 7455–9916; *P* = 0.001) during weeks 2–4. This remained stable during this 2-month follow-up period comparison, with a median TAb of 9916 (IQR 9660–9916) and 9916 (IQR 9095–9916) at 4–6 and 6–8 weeks, respectively (*P* = 0.801).

Although initially NaiveVax TAb remained lower up to 4 weeks after D1 in comparison with HospNoVax (TAb of 430; IQR 208–1241 versus 3414; IQR 311–5843; *P* < 0.001), the TAb was significantly higher after D2 (Supplemental Figure 1A). At 4–6 weeks after D1, the NaiveVax TAb was 1.2-fold higher than HospNoVax and 7.7-fold higher than OutPtNoVax (NaiveVax TAb: 7919 [IQR 6832–9339]; HospNoVax TAb 6683 [IQR 5573–8490]; OutPtNoVax TAb 1029 [164–2966]; *P* < 0.001) (Figure 3, Supplemental Figure 1A, and Supplemental Figure 2A).

### Qualitative antibody response during first 2 months in vaccinated versus SARS-CoV-2–infected individuals

#### Neutralizing antibody activity.

In all 3 cohorts, the neutralization activity gradually increased over time, albeit at different time scales (Figure 2B and Supplemental Figure 1B; note: neutralizing activity is inversely proportional to the %B/B0 in the figures). In the regression models, HospNoVax had a gradual increase in neutralizing activity up to 24 days postinfection with a change in SNAb of 5.537 %B/B0 per day (*P* < 0.001). The neutralization activity then began to wane, but the change was insignificant (mean 7.716%B/B0; coefficient = –0.51442; *P* = 0.055).

In comparison, NaiveVax displayed a slight lag in generating neutralizing activity after D1, with no changes from days 0 to 7 after D1 (coefficient 0.1476; *P* = 0.36). From days 7 to 30 after D1, the neutralizing activity increased at a rate of 4.304 %B/B0 per day (*P* < 0.001). Then, as seen with the TAb, there was loss of neutralizing activity after day 30 (*P* < 0.001) at a rate of 0.115%B/B0 per day.

In comparison, RecoVax had a much more dramatic increase in neutralization activity (Figure 2B). The neutralization activity improved at a rate of 9.381 %B/B0 in the first 8 days after D1 (*P* = 0.003), at which point the neutralization activity mildly began to wane at 0.008 %B/B0 per day (*P* = 0.005).

As with the TAb, RecoVax displayed consistently higher neutralizing activity across all time periods when compared with the other cohorts (Figure 3B, Supplemental Figure 1B, and Supplemental Figure 2B). In the first 2 weeks alone, the SNAb was 8.0- and 6.4-fold better than that of NaiveVax and HospNoVax, respectively (*P* < 0.001) (median SNAb %B/B0: RecoVax 12.48, IQR 1.14–69.84; NaiveVax 100, IQR 95.53–100; HospNoVax 80.07, IQR 31.77–100). After the second dose of the vaccine, at 4–6 weeks after infection/vaccination, the RecoVax SNAb neutralizing activity continued to remain higher than that of the NaiveVax, HospNoVax, and OutPtNoVax cohorts (*P* < 0.001) (Supplemental Figure 1B, Supplemental Figure 2B, and Figure 3).

Of note, NaiveVax SNAb at this 4- to 6-week time point had improved significantly and was at the levels found in HospNoVax (SNAb %B/B0 1.85, IQR 0.79–2.98; versus 1.19, IQR 0.58–4.22, respectively; *P* = 0.367). Furthermore, the NaiveVax SNAb neutralizing activity was 14.8-fold higher than the OutPtNoVax (SNAb %B/B0 NaiveVax 1.85, IQR 0.79–2.98; versus OutPtNoVax 27.55, IQR 8.44–63.48; *P* < 0.001) and remained 8.6-fold higher during the 6- to 8-week postvaccination period (SNAb %B/B0 NaiveVax 3.38, IQR 1.87–6.60; versus OutPtNoVax 29.03, IQR 9.05–55.25; *P* < 0.001) (Supplemental Figure 1B, Supplemental Figure 2B, and Figure 3B).

#### Antibody avidity.

Generally, over the initial 6-week time period, the strengthening of antibody avidity in any of the 3 cohorts was a gradual process, as would be expected (Figure 2C, Supplemental Figure 1C, and Supplemental Figure 2C; note: avidity is inversely proportional to the relative dR in the figures). Using regression models, we noted that the HospNoVax cohort avidity did not significantly change over time during its 61-day follow-up period (mean 8.998 × 10^–4^/s; coefficient = 0.0208 × 10^–4^; *P* = 0.162).

Probably because of the early production and then disappearance of IgM antibodies, NaiveVax appeared to display a period of avidity worsening with a dR change of 3.422 × 10^–6^/s per day (*P* = 0.02). However, at about 46 days postvaccination, avidity improved at a rate of 3.523 × 10^–6^/s per day (*P* < 0.001).

RecoVax consistently held a stronger avidity in comparison with the other cohorts (Supplemental Figure 1C, Supplemental Figure 2C, and Figure 3C). Its avidity slowly but consistently improved at a rate of 0.5 × 10^–6^/s per day. Its consistently stronger avidity was noted at multiple time points. For example, at 4–6 weeks after infection or D1 (Figure 3C), the median RecoVax avidity (median dR 4.24 × 10^–4^/s, IQR 4.323 × 10^–4^/s to 5.195 × 10^–4^/s) was nearly 2.5-fold higher (*P* < 0.001) than that of NaiveVax (4.24 × 10^–4^/s, IQR 4.323 × 10^–4^/s to 5.195 × 10^–4^/s), HospNoVax (4.24 × 10^–4^/s, IQR 4.323–5.195 × 10^–4^/s), and OutPtNoVax (4.24 × 10^–4^/s, IQR 4.323 × 10^–4^/s to 5.195 × 10^–4^/s). However, it should also be noted that the NaiveVax, HospNoVax, and OutPtNoVax held a similar avidity at all time points (e.g., *P* = 0.624 at 4–6 weeks).

### Comparison of antibody dynamics after D1 and D2 in RecoVax and NaiveVax up to about 6 months postvaccination

**Figure 4:**
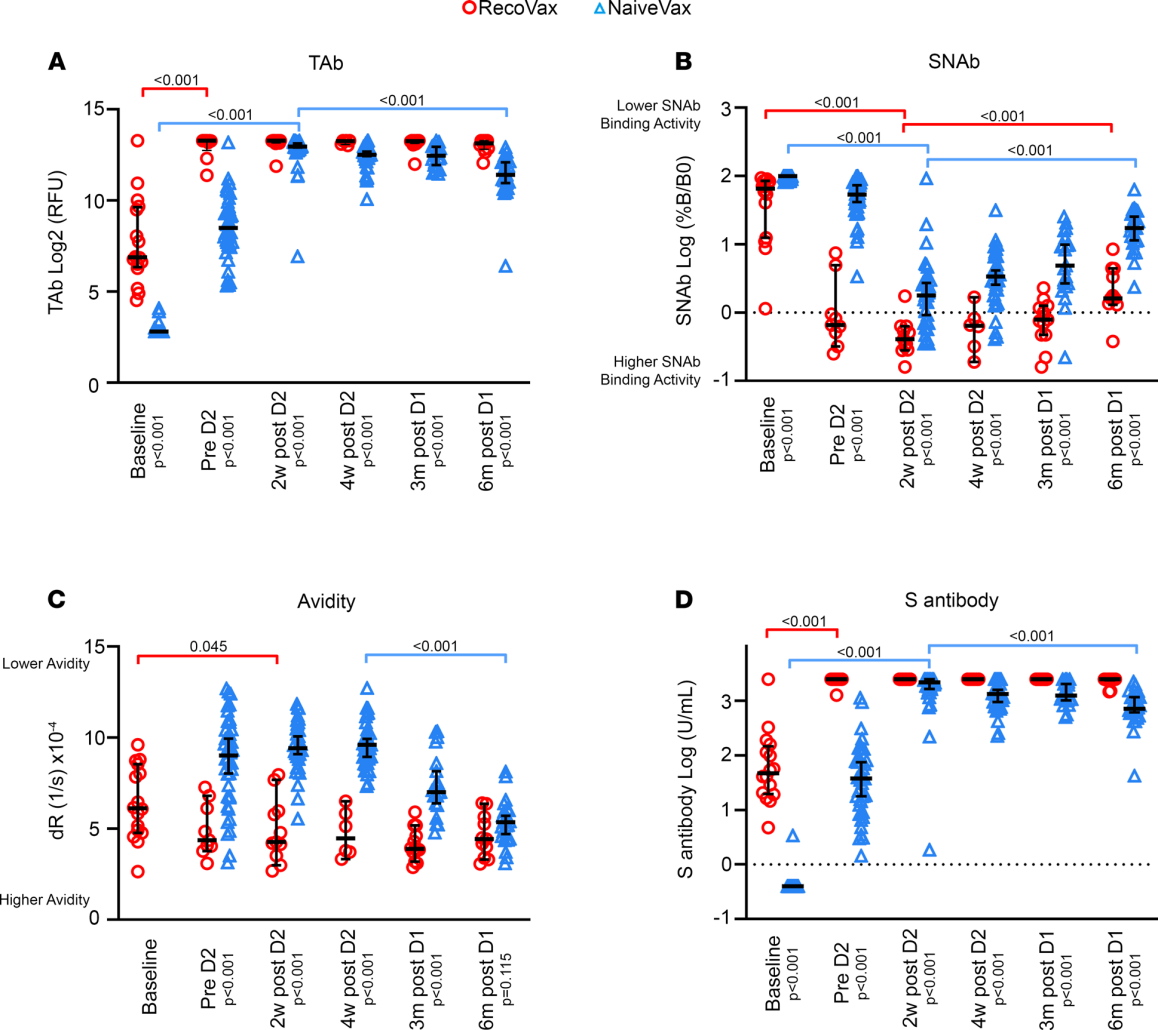
SARS-CoV-2 antibody profile of RecoVax and NaiveVax cohorts from baseline to about 6 months postvaccination. TAb antibody response (**A**), SNAb levels (**B**), avidity (**C**), and anti-S (**D**) of RecoVax individuals (red circle; total *n* = 66) and NaiveVax individuals (blue triangle; total *n* = 201) after the first (D1) and second (D2) doses of the vaccine. Comparisons were made at baseline (median 1 day; IQR 0–6 days after D1), prior to D2 (median 16 days after D1; IQR 15–21), approximately 2 weeks after D2 (median 35 days after D1; IQR 34–36), approximately 4 weeks after D2 (median 49 days after D1; IQR 49–52), approximately 3 months after D1 (median 100 days after D1; IQR 97–105), and approximately 6 months after D1 (median 168 days after D1; IQR 164–170). Horizontal black lines represent median values and whiskers represent 95% CI. Wilcoxon’s rank-sum was used for paired comparison while Kruskal-Wallis test was used for the comparison of 3 or more groups. S, spike.

Comparisons were made between the 2 vaccinated cohorts by analyzing the TAb levels, SNAb levels, and antibody avidity during key time periods in relation to the vaccine doses (Figure 4). The data were binned into (a) a baseline time period (median 1 day; IQR 0–6 after D1), (b) prior to D2 (median 16 days after D1; IQR 15–21), (c) about 2 weeks after D2 (median 35 days after D1; IQR 34–36), (d) about 4 weeks after D2 (median 49 days after D1; IQR 49–52), (e) about 3 months after D1 (median 100 days after D1; IQR 97–105) and (f) about 6 months after D1 (median 168 days after D1; IQR 164–170).

#### Quantitative antibody changes over time.

RecoVax had TAb already present at baseline (median 118 RFU; IQR 81.25–792 RFU), and these levels rapidly increased after D1 (9916 RFU; IQR 7154–9916; *P* < 0.001), prior to D2. Although there was a mild decrease in TAb at the ~6-month time point (8997 RFU; IQR 7179–9916), this was statistically insignificant (*P* = 0.698). The levels remained relatively unchanged throughout the study period.

This is in stark contrast to NaiveVax’s TAb, which had a more gradual increase in levels, with most individuals (40/41; 97.6%) not displaying positive TAb during the first week after D1. But all NaiveVax individuals did mount an antibody response in the following weeks prior to receiving D2, with a median TAb of 364.5 RFU (IQR 205.3–782.3). However, this was still 27-fold lower than the median TAb in RecoVax (*P* < 0.001). Maximal NaiveVax TAb levels were not achieved until 2 weeks after D2 (median 7919 RFU; IQR 7253–9170), but these TAb decreased by over 50% over time, with a median TAb 2706 RFU (IQR 1667–4511; *P* < 0.001) at about 6 months after D1, and were 3.3-fold lower compared with RecoVax (*P* < 0.001) at this time point (Figure 4A).

As a comparison substudy to an EUA platform, these results were confirmed by Elecsys Anti-SARS-CoV-2 S antigen assay, and similar patterns in the antibody response postvaccination were observed. RecoVax had detectable levels of the anti-S TAb prior to vaccination, and following D1 levels increased from a median baseline of 47.45 U/mL (IQR 19.59–148.7) to above the upper limit of detection (>2500 U/mL). The RecoVax cohort median remained at this level at about 6 months after D1; however, 3 individuals had levels below 2500 U/mL (median 2500 U/mL, IQR 2400 to >2500). In contrast, NaiveVax antibody levels gradually increased over time, with a median level of 37.77 U/mL (IQR 12.93–80.45 U/mL) prior to D2. These levels were boosted after D2 to a median of 2177 U/mL (IQR 1605 to >2500 U/mL) 2 weeks after D2 (*P* < 0.001). However, levels decreased to a median of 720 U/mL (IQR 565–1269; *P* < 0.001) at about 6 months after D2. Despite a robust increase after D2, the anti-S TAb levels consistently remained lower than those of RecoVax (*P* < 0.001 at all time points) (Figure 4D).

#### Neutralizing antibody changes over time in vaccinated patients.

In comparing the SNAb levels in NaiveVax and RecoVax, RecoVax displayed neutralizing capabilities at baseline, with median SNAb of 65.42 %B/B0 (IQR 19.47–84.59), which is 1.5-fold greater than NaiveVax (100%B/B0; IQR 96.09–100; *P* < 0.001). As described previously, the neutralization capability seen in RecoVax improved dramatically after D1, prior to D2 (0.6600 %B/B0; IQR 0.4150–2.955; *P* < 0.001). Although neutralizing capability remained unchanged through the ~3-month period (0.7900 %B/B0; IQR 0.4741–1.217; *P* = 0.144), at about 6 months after D1, neutralizing capability began to wane (1.610 %B/B0; IQR 1.359–4.424; *P* = 0.002).

Neutralizing activity was 81-fold lower in NaiveVax prior to administration of the second vaccine dose (median 53.57 %B/B0; IQR 31.29–77.95; *P* < 0.001). Although the neutralizing activity of NaiveVax also improved with time, it remained 5.2-fold lower than RecoVax at 4 weeks after D2 (3.380%B/B0; IQR 1.8015–6.603; *P* < 0.001), 6.1-fold lower about 3 months after D1 (4.867%B/B0; IQR 2.544–11.17; *P* < 0.001), and 10.8-fold lower about 6 months after D1 (17.35%B/B0; IQR 10.81–28.76; *P* < 0.001). Also of note, neutralization activity began to wane in NaiveVax at 4 weeks after D2 and continued to decrease at the ~6-month time point (*P* < 0.001) (Figure 4B).

#### Antibody avidity changes over time in vaccinated patients.

When comparing the avidity levels between RecoVax and NaiveVax cohorts, RecoVax had higher avidity levels prior to D2, twice those of NaiveVax (dR: 4.372/s [IQR 3.904–6.465] × 10^–4^/s versus 9.018/s [IQR 6.758–10.44] × 10^–4^/s; *P* < 0.001). This gap in avidity levels between the 2 cohorts was observed over all time periods up to about 3 months. At 4 weeks after D2, RecoVax still maintained a median avidity twice that of NaiveVax (dR: 4.463/s [IQR 3.623–6.000] × 10^–4^/s versus 9.605/s [IQR 8.773–10.29] × 10^–4^/s; *P* < 0.001) and 1.8-fold at 3 months postvaccination (dR: 3.889 [IQR 3.464–4.890] × 10^–4^/s versus 7.000 [IQR 6.335–8.380] × 10^–4^/s; *P* < 0.001). However, at about 6 months after D1, the gap in avidity closed with RecoVax avidity at 4.432 (IQR 3.390–5.642) × 10^–4^/s and NaiveVax avidity at 5.362 (IQR 4.509–5.977) × 10^–4^/s (*P* = 0.115) (Figure 4C).

## Discussion

The following conclusions can be drawn from our study. First, RecoVax individuals exhibited a rapid anamnestic SARS-CoV-2 TAb and anti-S antibody response within days after D1, and these levels persisted up to the ~6-month postvaccination follow-up period. Second, the antibody response did not further increase after the second vaccine dose in this population. This is in contrast to the other cohorts, where TAb levels never fully matched RecoVax and in some cases decreased. Third, neutralizing activity was present at baseline in RecoVax and remained significantly higher (Figure 2B) in RecoVax versus the other cohorts, reaching a maximal plateau after D1 with no significant change until the ~6-month follow-up period. However, at this point, RecoVax SNAb began to wane (*P* = 0.002) (Figure 2 and Figure 4B). Finally, RecoVax had twice the avidity of the other cohorts in the first 2 weeks after the immunizing event and sustained this level of avidity throughout the study follow-up period. Nonetheless, NaiveVax’s continued avidity maturation achieved a similar avidity level by about 6 months after D1. Overall, the results of our study build upon and contribute to a growing body of evidence that vaccination generates similar, if not superior, antibody levels to natural SARS-CoV-2 infection (9, 12, 15, 16, 26).

To our knowledge, this is the first longitudinal study to compare avidity in these 4 included study populations. Antibodies with low intrinsic avidity are initially produced in the early humoral immune response and require time to mature and strengthen. Thus, it was not unexpected to find that NaiveVax, OutPtNoVax, and HospNoVax generated antibodies with similarly lower avidity than those of RecoVax. Although it was expected that RecoVax at baseline would have significantly higher antibody avidity (Figure 2 and Figure 4C), it was notable that the antibodies induced by the vaccine showed robust avidity and that the rate of avidity maturation was greater in NaiveVax in comparison with RecoVax (ΔdR 0.03523 versus 0.005645 × 10^–4^/s per day), allowing NaiveVax avidity to match that of RecoVax at about 6 months after D1 (Figure 2C and Figure 4C) This observation, together with the knowledge that avidity continues to mature over time (23), warrants additional investigation on whether long-term avidity plays a clinically significant role in protection against SARS-CoV-2.

The immunological correlation of protection or thresholds required for vaccine efficacy against SARS-CoV-2 infection are not well defined (27). Although the FDA does not recommend monitoring antibody levels after SARS-CoV-2 vaccination (28), studies do attempt to correlate antibody levels to protection against the virus or disease when monitoring the immune response to vaccination, as antibody assays are generally convenient to perform. However, these may be nonmechanistic correlations and by no means absolute correlates (29). Recently, studies (30, 31) have begun to define the relationship between SARS-CoV-2 vaccine efficacy and neutralizing antibody titers by demonstrating significant correlation between vaccine efficacy and vaccine-induced neutralizing antibody activity. Neutralizing antibodies bind to viral targets and block entry into the cell, preventing infection of the cell with the virus. In this light, antibody avidity should not be discounted, as it is the binding strength of an antibody-antigen complex and could define the quality of the immune response (32). Although avidity is typically low immediately after infection or vaccination, it undergoes maturation over a period of months and could reflect a better functioning active antibody pool. Therefore, future studies should be attempted in correlating antibody avidity with vaccine efficacy as antibody avidity may be a reliable and stable long-term surrogate marker of immunological memory for infection (33).

Such studies could also address concerns about the persistence and protective strength of the antibodies generated by vaccination and natural infection (34–36). There may be concern over antibody levels in those with mild COVID-19 symptoms (i.e., OutPtNoVax) as this population has overall lower antibody levels in comparison with individuals with more severe symptoms (37) (i.e., HospNoVax) or vaccinated individuals (Figure 3). Unfortunately, this study did not have adequate long-term data for avidity regression modeling in the OutPtNoVax cohort, but early studies do reveal that avidity matures up to 1 year postinfection.(26). Together with the proper future correlative studies, it may become reassuring that avidity in OutPtNoVax, in HospNoVax, and in NaiveVax are similar (Figure 3C) and may continue to further strengthen over time, as demonstrated in NaiveVax, when avidity reached equivalent levels to that of RecoVax by about 6 months after D1 (Figure 4C).

Similar concerns over poor antibody production and protection against SARS-CoV-2 have arisen in at-risk populations, such as those with hematological malignancies or transplant patients (38–40). Although these at-risk individuals mount a poorer quantitative antibody response, it may be of interest to study their antibody avidity maturation profile and determine whether it could offer any protection against the virus.

### Limitations.

Our study has several limitations. First, selection bias may exist in both the prospective and retrospective studies. As the HospNoVax and OutPtNoVax specimens in the study were retrospectively collected, the study was reliant on preexisting data and remnant specimens. During this early time period of the pandemic in New York City, most hospitalized individuals were older, had more severe symptoms, and were predominately male (41), which was reflected in our cohort (median age 67.5 years, IQR 54.0–77.0; 66% male). The prospective study participants were younger, approaching or at middle age (overall median age 44.4 years, IQR 33.6–57.4), and predominantly female (74%). Indeed, the better population for comparison is OutPtNoVax, but there were insufficient data to perform a direct comparison at those similar time points.

Additional bias may exist with the prospective vaccination study volunteers, as study participants were health care workers (eligible for the vaccine in late December 2020 to January 2021), possibly reflecting a study population with fewer comorbidities. Together with the small sample size (*n* = 60), these results may not represent those of the general population (42, 43).

Second, given the exploratory nature of the study and limited sample size, post hoc adjustments were not performed. This small study size also prevented multivariate analysis to look for possible confounders between the cohorts. For example, a previous study found SNAb to be age associated in SARS-CoV-2–infected individuals (42). The association of age with TAb, SNAb, and avidity was explored in this study, but no association was found, likely due to the predominately middle-aged cohort in this study.

Third, the time period between symptom onset and antibody testing in HospNoVax is arguably not an exact time equivalent to the time period of antibody testing postvaccination, as the incubation period and symptom onset postinfection can be up to 14 days (24). However, it has been reported that the median incubation period is 5.1 days (25), and this was added to the OutPtNoVax and HospNoVax’s time since symptom onset in an attempt to overcome lead time bias. Additionally, the follow-up time for this cohort was limited compared with the vaccinated cohorts.

Finally, this study focused on the humoral response to the SARS-CoV-2 vaccine, as vaccine development strategies are designed to maximize this response. However, the T cell compartment also plays a major role in the immune response (44). SARS-CoV-2 infection has been shown to induce the T cell immune response, which plays a major role in preventing severe disease (45, 46). Although this is beyond the scope of this study, studying the interplay between the T cell response and humoral response may also provide important insights into better correlative studies for vaccine effectiveness.

### Conclusions.

Our data suggest that 2 doses of the mRNA vaccine are warranted in NaiveVax individuals to achieve a similar early antibody response to RecoVax individuals. Individuals with mild COVID-19 symptoms (OutPtNoVax) overall maintained lower antibody levels compared with the vaccinated cohorts, especially warranting vaccination despite prior infection. Furthermore, as the vaccine elicited a maximal antibody response after only 1 vaccine dose in RecoVax, 1 dose may be sufficient in this population. Although longer term longitudinal studies are required, the persistent TAb and avidity in this population may support a single-dose vaccination strategy. This would be a resource-conscious solution to help address global vaccine shortages. Monitoring individuals for antibody titers long term (as is done with the hepatitis B or MMR vaccines), as well as monitoring neutralizing activity and avidity (23), may be prudent in determining the vaccine efficacy and the need for future booster vaccinations.

## Methods

### Sources of serum specimens and data acquisition

The standard practice for serum collection and storage in the clinical laboratories involves collecting venous blood into a serum separator tube, allowing the specimen to fully clot, and then centrifuging the specimen (1500*g* for 7 minutes at room temperature) within 2 hours of collection to separate cells from serum. Patient and study participant serum samples after routine clinical testing were stored at –80°C until further analysis was performed.

#### Retrospective study of COVID-19 outpatients and hospitalized patients.

The retrospective study included a cohort of 122 adult patients who presented to the ED and were subsequently hospitalized at NYP/WCMC from March 8, 2020, to April 7, 2020. This cohort was described in a previous assay validation study (23). All patients in this cohort tested positive for SARS-CoV-2 by RT-PCR within 1 day of the ED visit. In total, 317 remnant serum samples were collected and frozen during the hospitalization for future analysis.

Another cohort from this retrospective study included 160 convalescent COVID-19 patients who were seropositive in the outpatient setting at NYP/WCMC from April 30, 2020, to May 20, 2020. All participants in this study were adult, nonpregnant patients who were not hospitalized (previously or at the time of antibody testing) due to SARS-CoV-2 infection. In total, 160 remnant serum samples were collected and frozen for this future analysis.

Demographic data and date of symptom onset were collected from the electronic medical record for both cohorts (Allscripts).

#### Prospective study of vaccinated individuals.

Sixty-one health care workers at NYP/WCMC vaccinated with 2 doses of the BNT162b2 SARS-CoV-2 mRNA vaccine (Pfizer) were included in this study (Figure 1). Participants were asked to donate blood samples for serologic analysis within a week of the first dose of the vaccine (D1), approximately 2 weeks and 4 weeks after each vaccine dose, and approximately 3 and 6 months after D1. Although not all the 61 participants provided samples at all time points, all specimens were considered for analysis in this study, as indicated in the figures and tables. A total of 326 serum samples were prospectively collected December 31, 2020, to July 1, 2021 (Figure 1).

An additional 7 participants from the NYP-WELCOME study (47) were included in the prospective aspect of this study. Forty-three specimens had been collected from this cohort during the time period December 11, 2020, to July 6, 2021, and frozen in the Weill Cornell Institutional Biorepository Core for future analysis (Figure 1).

### SARS-CoV-2 total RBD antibody, SNAb assay, and avidity assays

Further information can be found in Supplemental Methods. The SARS-CoV-2 total RBD antibody (TAb), surrogate neutralizing antibody (SNAb), and avidity were used to measure serum antibody levels on the TOP-Plus (Pylon 3D analyzer; ET Healthcare) and were previously described (23).

The TAb assay measures the overall interaction between SARS-CoV-2 antibodies and the RBD of the virus S protein, with a readout of sample RFU.

SNAb assay is a competitive binding assay, based on the anti–SARS-CoV-2 antibody–mediated inhibition of the interaction between the angiotensin-converting enzyme 2 (ACE2) receptor protein and the RBD. The assay readout is the percentage of RBD-ACE2 binding (%B/B0; [sample RFU/negative control RFU] × 100%), which is inversely associated with neutralizing activity. SNAb assay was previously shown (23) to correlate well with 2 established SARS-CoV-2 virus neutralization tests (plaque reduction neutralization test and pseudo virus neutralization test) and was used in this study to evaluate the neutralization activity of the antibodies generated postvaccination and postinfection in ED COVID-19 patients in this study.

The avidity assay provides the calculated relative dR (1/s). This measurement is inversely associated with antibody avidity. A higher intrinsic binding strength of a paratope to RBD or addition of paratopes to the antibody structure results in a higher binding strength, which results in a lower dR of the antibody-RBD pair. The assay had good correlation with the Bio-Layer Interferometry avidity assay, another assay used for measuring antibody avidity (23).

### The Roche Elecsys Anti-SARS-CoV-2S and N antigen assays

The Elecsys Anti‑SARS‑CoV‑2S and Anti-SARS-CoV-2 electrochemiluminescence immunoassays (Roche Diagnostics) were used to detect antibodies against SARS-CoV-2 S RBD and the N antigen, respectively, in the serum samples. These were performed on the Roche Diagnostics Cobas e411. These assays received EUA from the FDA. The Elecsys Anti‑SARS‑CoV‑2S assay was used for comparison with the TOP-TAb assay. The Elecsys Anti-SARS-CoV-2 assay was used in identifying or confirming previously infected individuals in the vaccinated cohort.

### Statistics

The trend of antibody level over time was described by applying Muggeo’s method of estimating regression models with unknown break-points to estimate the changing time points of the trends (48). A linear mixed effect model was fitted for each segment of time based on estimated breakpoint to show the trend for the time period between breakpoints. As indicated, coefficients and *P* values from regressions were reported. To visualize the trend, trajectories were plotted via a smooth line with loess method for each group. Only data up to day 61 postinfection were available for this analysis in HospNoVax, and comparisons were only performed up to this time period.

Wilcoxon’s rank-sum and signed-rank tests were used between numerical variables and paired comparison, respectively. Kruskal-Wallis test was used for the comparison of 3 or more groups. Bivariate associations between outcome variables and clinical parameters were evaluated using Fisher’s exact test or χ^2^ test, as appropriate. Descriptive data were presented as median with IQR unless otherwise specified. *P* < 0.05 was considered significant.

Analyses were performed in statistical programming language R version 4.0.2 (2020-06-22) or in GraphPad Prism Version 9.1.2 (GraphPad Software).

### Study approval

The retrospective (IRB 20-03021671) and prospective (IRB20-11022929; IRB 20-04021831) studies in this manuscript were performed at NYP/WCMC with approval by the IRB of Weill Cornell Medicine. Written informed consent was collected from the participants in the prospective arms of this study. Informed consent was waived by the IRB for the retrospective arms of this study.

## Author contributions

SERB was responsible for conceptualization, investigation, supervision of the project, data analysis, and writing the manuscript; JKY was responsible for sample collection, performing serology experimentation, and editing the manuscript; and order of first authorship was agreed upon by SERB and JKY in light of the conceptualization and writing of the manuscript by SERB. AS was responsible for sample collection and performing serology experimentation; YQ for statistical analysis and editing the manuscript; SR for data collection, data collection design, and general administrative support; PDB for recruitment efforts; YH for sample collection; HSY for conceptualization and editing the manuscript; FSA and QHM for conceptualization and editing the manuscript; YS for conceptualization; AC for editing the manuscript; EG and SCF for design and implementation of the NYP-WELCOME study and editing of the manuscript; MMC for supervision of project and editing the manuscript; and ZZ for conceptualization, investigation, supervision of the project, data analysis, and editing the manuscript.

## Supplementary Material

Supplemental data

## Figures and Tables

**Table 1:**
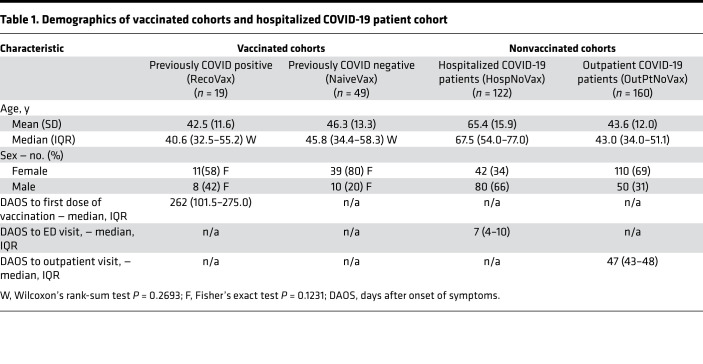
Demographics of vaccinated cohorts and hospitalized COVID-19 patient cohort

## Version 1 09/09/2021

In-Press Preview

## Version 2 10/22/2021

Electronic publication
